# Effects of the Use of Different Temperature and Calcium Chloride Treatments during Storage on the Quality of Fresh-Cut “Xuebai” Cauliflowers

**DOI:** 10.3390/foods11030442

**Published:** 2022-02-02

**Authors:** Bingyu Mu, Jianxin Xue, Shujuan Zhang, Zezhen Li

**Affiliations:** 1 College of Agricultural Engineering, Shanxi Agricultural University, Jinzhong 030801, China; 13633544838@163.com (B.M.); Z15035658426@163.com (S.Z.); 2 College of Food Science and Engineering, Shanxi Agricultural University, Jinzhong 030801, China; l19834545023@163.com

**Keywords:** fresh-cut cauliflower, storage period, calcium chloride, temperature, factor analysis, quality

## Abstract

This study revealed the effect of the use of different temperature and calcium chloride (CaCl_2_) treatments on the storage quality of fresh-cut “Xuebai” cauliflowers. Fresh-cut “Xuebai” cauliflowers were soaked with 2% CaCl_2_ solution at different temperatures. The change in the firmness, color, and ascorbic acid (ASA), total glucosinolates (TGLS), polygalacturonase (PG), and lipoxygenase (LOX) content of fresh-cut “Xuebai” cauliflowers during the cold storage period was assessed. In addition, the sensory quality was also evaluated. The results show that the combined treatments with CaCl_2_ at different temperatures could effectively maintain the storage quality of fresh-cut “Xuebai” cauliflowers. Then, a method based on factor analysis with comprehensive quality evaluation was proposed. A factor analysis with a principal component analysis (PCA) was conducted on nine indicators of cauliflowers. Two principal components were extracted with a cumulative contribution rate of 97.513%. The results demonstrated that the treatment with the best fresh-keeping effect of cauliflowers in storage was the combination treatment at 40 °C with 2% CaCl_2_ solution, while the optimal storage period was 15 days.

## 1. Introduction

Brassica vegetables are important components of a healthy diet and can help to prevent diseases such as diabetes, cardiovascular disease, and certain cancers; thus, they have attracted the attention of researchers [1,2,3]. Among cruciferous vegetables, cauliflowers (*Brassica oleracea* L. botrytis) are important components of human diets and have large economic benefits and yields around the world. The demand for cauliflowers has risen sharply due to their high nutritional value and high levels of many bioactive compounds, including glucosinolates, phenolic compounds, and ascorbic acid [4,5].

Fresh-cut fruits and vegetables are packed and sold after fresh raw materials have been selected, cleaned, peeled, and sliced [6]. Nowadays, with changes in lifestyles and consumption habits, the popularity of fresh-cut cauliflowers has risen among consumers due to their ideal nutritional value, excellent sensory qualities, and health-related benefits for the body [7]. As a basic processing method, cutting will cause browning, odor emission, tissue softening, and a loss of nutritional quality in fresh-cut cauliflower, thus shortening its storage time [8]. As a divalent cation nutrient element, calcium (Ca^2+^) plays a significant role in delaying aging and improving antioxidant capacity in the cell wall and membrane structure. During the cross-linking process between Ca^2+^ and carboxyl groups in pectin, a structure called “egg box” is formed. This helps to increase the strength of the cell wall and the firmness of the tissue structure [9].

Calcium chloride (CaCl_2_) as a preservative and curing agent has been widely used in the preservation of fresh-cut fruits and vegetables, as it can effectively inhibit the occurrence of diseases, inhibit ethylene production, and delay the aging of fruits and vegetables [10,11]. Aghdam et al. (2013) found that postharvest CaCl_2_ treatment had a positive effect on the antioxidant capacity and DPPH free radical scavenging capacity of cornelian cherry (*Cornus mas*) fruit. At the same time, the content of active substances such as anthocyanins and ascorbic acid was increased [12]. Zhang et al. (2019) reported that treatment with 2% CaCl_2_ could reduce the browning of “Nanguo” pear peel during cold storage, maintain a high hardness and polyphenol content, and inhibit the gene expression of phospholipase D and polyphenol oxidase while reducing their activity [13].

In recent years, heat treatment has attracted researchers’ attention because of its advantages in terms of safety, lack of contribution to pollution, and easy means of operation. Through heat treatment, the incidence of chilling injury symptoms in fruits and vegetables can be reduced; the occurrence of respiratory peaks can be delayed; losses of water can be reduced; the ripening, decay and yellowing of fruits and vegetables within the storage period can be controlled; the postharvest quality of fruits and vegetables can be maintained, and their shelf life can be prolonged [14]. As a common method of physical pretreatment for fruits and vegetables, cold treatment can reduce the tissue temperature of fruits and vegetables within a short timeframe, cause them to experience low-temperature stress, induce stress resistance, maintain the balance of active oxygen metabolism in fruits, protect the integrity of the cell membrane structure, reduce enzyme activity, delay fruit aging, and maintain the nutritional quality of fruits and vegetables to the greatest extent possible [15]. Duarte-Sierra et al. (2017) reported that heat treatment can lower the respiratory efficiency of broccoli, maintain a good chemical composition, delay the rate of yellowing of the curd, and lead to a significantly higher content of chlorophyll and glucosinolate than is present in untreated broccoli [16]. Yang et al. (2016) found that CaCl_2_ treatment can increase glutamine biosynthesis in broccoli, promote myrosinase (MYR) gene expression and activity, and improve the total antioxidant capacity and DPPH scavenging capacity [17]. Grzegorzewska et al. (2009) found that, during the storage period, broccoli treated with ice water had a lower rooting rate, lower loss of tightness, and minor color change [18].

Some research reports have shown that CaCl_2_ treatment combined with other methods is a good method of fruit and vegetable preservation. Wang et al. (2014) reported that the combined treatment with CaCl_2_ and cold water at 0 °C can inhibit the respiration rate of sweet cherries, help them maintain a brighter luster, enhance their antioxidant system, and reduce the peroxidation of membrane lipids. In the end, the authors achieved their goal of reducing the rate of decay of cherry fruit and delaying its senescence speed [19]. Supapvanich et al. (2012) suggested that the use of hot CaCl_2_ dips at 40 °C could help to maintain the postharvest quality of fresh-cut sweet leaf bush, delay the decrease in chlorophyll content, delay yellowing, and help to maintain a high nutritional quality, especially with regard to the activities of total phenols, flavonoids, and antioxidant enzymes [20].

Unfortunately, there has been no related exploration of the change in the preservation quality of fresh-cut “Xuebai” cauliflowers through combined treatment with temperature and CaCl_2_. Therefore, this study aimed to select the optimal temperature of CaCl_2_ solution for the preservation of fresh-cut “Xuebai” cauliflower florets at 4 °C. The effects of CaCl_2_ solutions of different temperatures on the color value, firmness, ascorbic acid content (ASA), total glucosinolates content (TGLS), and polygalacturonase (PG) and lipoxygenase (LOX) activities during storage of fresh-cut “Xuebai” cauliflower florets were studied. By measuring multiple indicators, conducting sensory evaluation (SE), combining principal component analysis and factor analysis, and sorting out the scores, the most suitable compound treatment method for fresh-cut “Xuebai” cauliflower preservation was screened. This study provided theoretical support for the research and development of storage and preservation technology for fresh-cut “Xuebai” cauliflower florets and the extension of shelf life.

## 2. Materials and Methods

### 2.1. Plant Material and Treatments

“Xuebai” cauliflower (*Brassica oleracea* L. botrytis) was picked in Juxin Agricultural Park (112.49 N and 7.39 E) in Taigu, Shanxi Province of China. After picking, the cauliflower was precooled by vacuum in the field, put into a foam box with small ice cubes, and transported to the laboratory. The selected cauliflower samples were similar in size and without any diseases, insect pests, or mechanical damage. We cut cauliflower into small flower balls (about 50 g) of the same size with a knife sterilized in 0.1% sodium hypochlorite solution, soaked the flower balls in 0.1% sodium hypochlorite solution for 5 min, rinsed them with sterile water, and took them out to dry.

After drying, 21 samples were separated from the freshly cut florets and analyzed. A total of 420 fresh-cut cauliflower samples were selected and divided into four groups (each with 105 samples); then, the following treatments were performed: (1) T20 °C-CaCl_2_ 0%: immersed in water at 20 °C for 10 min; (2) T0 °C-CaCl_2_ 2%: immersed in 2% CaCl_2_ solution at 0 °C for 10 min; (3) T20 °C-CaCl_2_ 2%: immersed in 2% CaCl_2_ solution at 20 °C for 10 min; (4) T40 °C-CaCl_2_ 2%: immersed in 2% CaCl_2_ solution at 40 °C for 10 min. Then, the fresh-cut “Xuebai” cauliflower florets were controlled in a drain basket, dried, and put into a 0.04 mm PE fresh-keeping bag at a constant temperature of 4 °C and a 90% constant humidity for 16 days. For each treatment, we randomly selected 21 samples (a total of 84 samples) for repeated experiments at 0, 3, 6, 9, 12, and 15 days. Then, we determined the quality index and performed a sensory evaluation.

According to the research of Grzegorzewska et al. (2009) and Xue et al. (2021), the temperature setting, number of storage days, and CaCl_2_ solution concentration of this experiment were determined [18,21].

### 2.2. Sensory Quality Scores

After each sampling, a group of volunteers (*n* = 9) consisting of male and female students were asked to evaluate the following sensory attributes: appearance, aroma, flavor, texture, and overall acceptability. These volunteers received professional training before the assessment and agreed on the assessment criteria. The sensory quality scores for cauliflower ranged from one to nine and were calculated using a weighted method. A score of nine meant the sample was excellent (the color was very good, the flower ball organization was tight, the fragrance was good, and it was not decayed); a score of seven meant it was good (the color and luster were good, tissue, the fragrance was light, and less than one twentieth of the flower buds had spots); a score of five was medium (the color and luster were acceptable, the flower balls were slightly soft, the central organization was loose, there was some fragrance, and one-twentieth to one-fifth of the flower buds had spots); a score of three was poor (the color and luster were not good, more than half of the flower balls were wilted, there was a slight peculiar smell, and one-fifth to one-half of the flower buds had spots); a score of one was extremely poor (the color was very bad, most of the bulbs were wilting, there was a rancid smell, and more than half of the flower buds had spots) [22,23].

### 2.3. Firmness Measurement

The firmness of the cauliflower floret was measured by the TMS-PRO food physical property analyzer (FTC, Sterling, VA, USA). The cylinder probe used was P / 2 n, the initial speed was 5 mm/s, the test speed was 1 mm/s, the retraction speed was 5 mm/s, the interval between two extrusions was 2 s, the minimum perception force was 0.4 N, and the extrusion depth was 4 mm. The firmness of each sample was measured three times, and the average value was calculated. The maximum firmness value obtained at the first compression was taken as the firmness value of the sample.

### 2.4. Color Measurement

Referring to the research of Yan et al. (2020), at each sampling time point, the L*, a*, and b* values on the surface of the cauliflower florets were measured using a CR-400 color meter (Konica Minolta, Tokyo, Japan) [23]. Three data points were randomly measured at the top bulge of each sample, and the average value was taken as the final result.

### 2.5. Ascorbic Acid Content Measurement

Cauliflower samples with weights of 1 g were ground and fixed to 50 mL; the content of ascorbic acid (ASA) was determined by titration with 2-6-dichloro-indophenol. When the ASA was completely oxidized, a drop of the dye caused the oxalic acid solution to immediately appear light pink. This change in color was the end point of titration. According to the titration standard of 2-6-dichloro-indophenol solution, the content of ascorbic acid in cauliflower florets can be calculated. The specific operation method used was based on the method of Chen et al. (2018) [24].

### 2.6. Total Glucosinolates Measurement

Based on the principle that total glucosinolates (TGLS) are hydrolyzed into glucose by myrosinase (MYR) in cauliflower, the content of TGLS was determined by spectrophotometry. A standard solution of 3-5 dinitrosalicylic acid and glucose was prepared, and the glucose standard curve was drawn. Two 0.5 g grinding samples were accurately weighed and placed in 25 mL calibration tubes, and to each, 0.1 g of sodium fluoride was added. Then, we added 20 mL of boiling water to a test tube, heated it immediately until it boiled, and maintained it there for 10 min. In the other test tube, we added 20 mL of distilled water at 36 °C and kept it in a water bath at 37 °C for one hour. The TGLS was hydrolyzed under the action of MYR. Then, we heated the sample to boiling and kept it there for 10 min. We added six drops of neutral lead acetate to each of the two test tubes, distilled water was added until the solution reached a total of 25 mL, and 0.5 mL of filtrate was used to determine its absorbance.

### 2.7. Polygalacturonase Measurement

The determination of polygalacturonidase (PG) was performed using visible spectrophotometry. The galacturonidase produced by PG hydrolysis reacted with DNS reagent to produce a brownish red substance with a characteristic absorption peak at 540 nm. The pectinase activity was calculated by measuring the change in the absorbance value at 540 nm.

We weighed 0.1 g of cauliflower, added 1 mL of extract, ground it in an ice bath, centrifuged it for 10 min at 4 °C with sixteen thousand revolutions, diluted the supernatant five times, and followed the steps of the determination instructions (Soleibao Technology Co., Ltd., Beijing, China).

### 2.8. Lipoxygenase Measurement

The presence of lipoxygenase (LOX) was determined by ultraviolet spectrophotometry (Soleibao Technology Co., Ltd., Beijing, China); LOX can catalyze the oxidation of linoleic acid, and the oxidation product had a characteristic absorption peak at 234 nm. The increasing rate of 234 nm absorbance was measured to calculate the LOX activity.

### 2.9. Statistical Analysis

For this experiment, we adopted a completely random design. A one-way analysis of variance (ANOVA) was performed on the quality indicators. All indicators were repeatedly measured three times (each repetition included 21 samples), and the results were expressed as mean ± standard deviation (SD). We used the SPSS 17.0 software and Origin 19 software for data analysis and the creation of graphs.

## 3. Results and Discussion

### 3.1. Effect of Different Treatments on the Quality of Fresh-Cut “Xuebai” Cauliflower Florets

#### 3.1.1. Volunteer Group Sensory Evaluation

**Figure 1:**
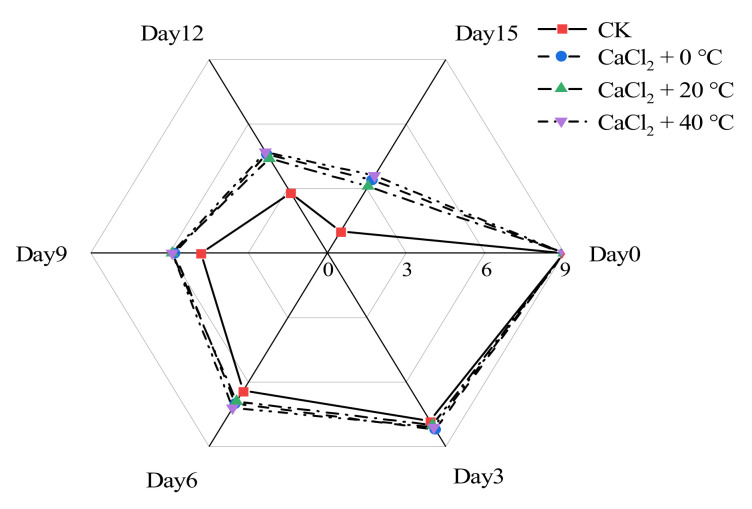
Sensory evaluation of fresh-cut “Xuebai” cauliflower florets given different treatments during storage.

The most intuitive indicator that describes the quality of cauliflower and reflects its commercial value is sensory evaluation (SE) [25]. It can be seen from Figure 1 that the sensory score of fresh-cut “Xuebai” cauliflower florets declined during the storage period. The sensory score of the control check (CK) was always lower than that of the other treatment groups. It can be seen from Figure 1 that from 0 d to 6 d, the sensory scores of the four groups of fresh-cut “Xuebai” cauliflower florets did not differ greatly. However, as the storage period grew longer, the decline in the scores of the CK group accelerated. On the 15th day, the CK group had the lowest score. Samples from this group scored only one point, indicating that they had lost their commercial value. These samples received significantly different scores from those of the groups treated with CaCl_2_ (*p* > 0.05). In general, the effect of the CaCl_2_ + 40 °C treatment was slightly better than that of the CaCl_2_ + 0 °C and CaCl_2_ + 20 °C treatment groups. Our results showed that treatments with CaCl_2_ can effectively maintain the marketability of cauliflower.

#### 3.1.2. Color

The color of cauliflower is very important for evaluating its value and quality. However, fresh-cut cauliflower will brown and rot in the postharvest storage process, which is visually manifested as a color change [26]. On the zeroth day of storage, the color values L*, a*, and b* of fresh-cut “Xuebai” cauliflower florets were 70.265 ± 0.96, −1.558 ± 0.67, and 9.61 ± 0.35 in sequence. In Table 1, the effects of storage time and treatment methods on the lightness L* value (light or dark), a* value (−green to + red), and b* value (−blue to + yellow) are compared. The results show that the lightness of all samples (CK and treated samples) decreased as the storage time grew longer, while the a* and b* values increased over the course of the storage period [27].

After a prolonged period of storage, the L* values of CaCl_2_ treated fresh-cut “Xuebai” cauliflower florets were significantly different from those of the CK group on the 12th and 15th day, indicating that CaCl_2_ can effectively inhibit the browning of fresh-cut “Xuebai” cauliflower florets and decrease the appearance of spots on the flower bud. Throughout the whole storage period, the L* value of fresh-cut “Xuebai” cauliflower florets treated with CaCl_2_ + 40 °C was higher than that of the other three groups. On the 15th day, there were significant differences between cauliflower florets from the CaCl_2_ + 40 °C treatment group and those from the other three groups (*p* < 0.05). In addition, the a* and b* values for cauliflower florets given CaCl_2_ + 40 °C treatment were the lowest, indicating that CaCl_2_ + 40 °C treatment can effectively prevent fresh-cut “Xuebai” cauliflower florets from turning yellow and browning. It can be seen that the CaCl_2_ + 40 °C treatment is better able to maintain the quality of fresh-cut “Xuebai” cauliflower florets.

Consistent with our research results, Zhang et al. (2014) [28] found that after heat treatment, the color value of the heat treatment group was better than that of the other treatment groups [28]. Suzuki et al. (2005) reported that heat treatment can reduce the rate of yellowing of cauliflower [29]. Supapvanich et al. (2012) reported that soaking freshly cut beets with hot CaCl_2_ solution can effectively lower the activity of chlorophyll-decomposing enzymes, reduce the loss of chlorophyll and protein in vegetables, delay the loss of greenness, and reduce the rate of yellowing [20].

#### 3.1.3. Firmness

**Figure 2:**
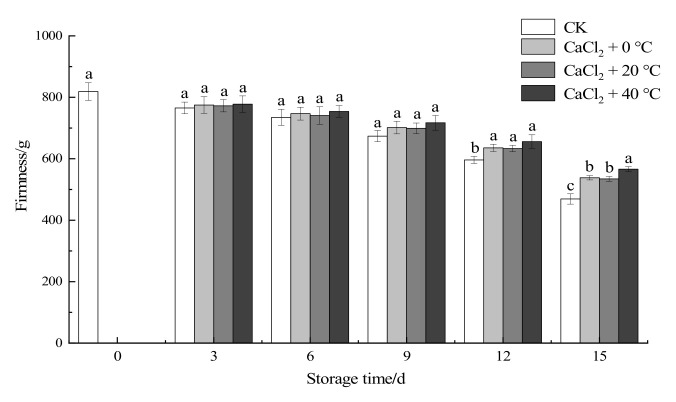
Effect of the different treatments on the firmness of fresh-cut “Xuebai” cauliflower florets. Error bars indicate standard deviation (SD); the statistical significance between the four groups that passed the Tukey significance test is expressed in different lowercase letters, and the significance level is *p* < 0.05.

In the process of postharvest storage and consumption, firmness is always an important indicator for evaluating the quality of cauliflowers [30]. In this study, the combined treatment with temperature and CaCl_2_ had a significant effect on the firmness of fresh-cut “Xuebai” cauliflower florets during their period of storage at 4 °C (*p* < 0.05). From Figure 2, there was no significant difference between the treatment groups and the CK group throughout the early and middle stages of storage (*p* < 0.05). However, after storage for a longer period of time, the rate of decrease in the firmness of fresh-cut “Xuebai” cauliflower florets given treatment was significantly lower than that of the CK group (12th and 15th days). By the 15th day, there were significant differences between the CK group and the three treatment groups (*p* < 0.05), indicating that the combined treatment was able to maintain the firmness more effectively and reduce the softening of the “Xuebai” cauliflower florets. The difference between the CaCl_2_ + 0 °C and CaCl_2_ + 20 °C treatment groups was not significant (*p* > 0.05), but the CaCl_2_ + 40 °C treatment group showed a significant difference from the other two groups (*p* < 0.05). A comprehensive comparison showed that the CaCl_2_ + 40 °C treatment was better able to maintain the firmness of fresh-cut “Xuebai” cauliflower florets.

Duarte-Sierra et al. (2017) studied the effect of heat treatment on the quality of broccoli in storage. The results showed that the use of different temperature treatments can lead to the formation of a high initial CO_2_ content, delay yellowing, maintain a higher level of firmness, and lead to a lower weight loss rate of broccoli [16]. Some enzyme activities relating to hydrolyzing cellulose and pectin increased, dissolving the middle gum layer, loosening the structure of the cell wall, and thus causing the softening of cauliflowers over the storage period [31]. The reason for the rapid decrease in the hardness value was the increase in the rate of metabolism, which is related to aging. Wang et al. (2014) showed that calcium pectate was formed after treatment with CaCl_2_, which increased the rigidity of the cell wall and reduced the content of PG, pectin methyl esterase (PME), B-galactosidase (b-Gal), etc. These substances are located in the middle layer of the cell wall and are closely related to the activity of enzymes related to fruit softening. In addition, calcium can also fix water and maintain the stability of the pressure between cells, delaying the appearance of softening [19].

#### 3.1.4. Ascorbic Acid Content

**Figure 3:**
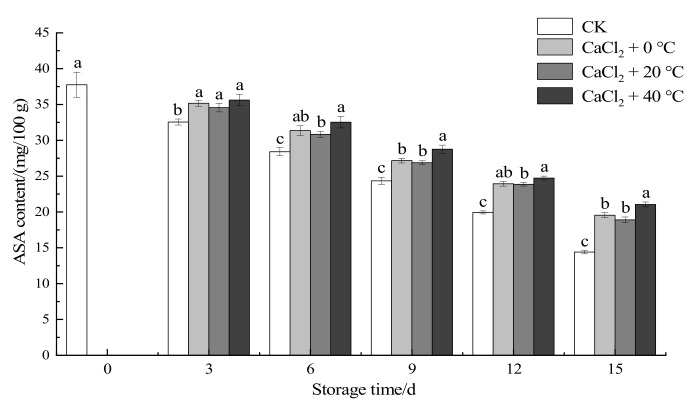
Effect of the different treatments on the ASA content of fresh-cut “Xuebai” cauliflower florets. Error bars indicate standard deviation (SD); the statistical significance between the four groups that passed the Tukey significance test is expressed in different lowercase letters, and the significance level is *p* < 0.05.

As shown in Figure 3, with the extension of the storage period of fresh-cut “Xuebai” cauliflower florets, the ASA content in the CK group and treatment groups showed a downward trend. However, the temperature and CaCl_2_ treatment effectively delayed the decrease in the ASA content. On the 15th day, there was a significant difference between the ASA value of fresh-cut “Xuebai” cauliflower florets given the combined treatments and that of florets in the CK group (*p* < 0.05). Additionally, there was a significant difference between the cauliflowers given the CaCl_2_ + 40 °C treatment and those in the other two treatment groups (*p* < 0.05). It can be seen that the CaCl_2_ + 40 °C treatment more effectively inhibited the decomposition of ASA during the storage period of fresh-cut “Xuebai” cauliflower florets.

Many fruits and vegetables are rich in ASA. As is widely known, ascorbic acid is an important antioxidant for the healthy growth of the human body. Ribeiro et al. (2020), in a study on red pomegranate, and Morteza Soleimani Aghdam et al. (2013), in a study on cornelian cherry fruit, reported the positive effect of calcium treatment on delaying the decrease in ASA content [12,32]. Naser et al. (2018), in a study concerning the quality changes in persimmons, showed that the use of a combined calcium and heat treatment can effectively decrease the reduction in ASA. Calcium treatment can act as a signal to activate the antioxidant system inside the cell; calcium treatment can reduce the rate of free radical degradation at the genetic level and reduce the consumption of ASA [33]. Aguayo et al. (2015) reported that heat treatment was used as an auxiliary method to help increase the absorption of ASA in apple tissues. Combined with calcium treatment, it can delay the decrease in ASA content and improve the quality of apples in storage [34].

#### 3.1.5. Total Glucosinolates Content

**Figure 4:**
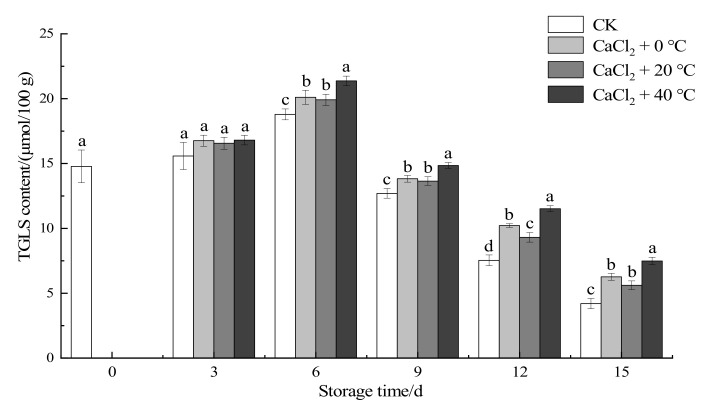
Effect of the different treatments on the TGLS content of fresh-cut “Xuebai” cauliflower florets. Error bars indicate standard deviation (SD); the statistical significance between the four groups that passed the Tukey significance test is expressed in different lowercase letters, and the significance level is *p* < 0.05.

Figure 4 shows that during the storage process, the total glucosinolates (TGLS) content of fresh-cut “Xuebai” cauliflower florets followed a trend of increasing first and then decreasing; these results are the same as those of Wei et al. (2016), Duarte-Sierra et al. (2017), and Xue et al. (2020) [21,35,36]. The TGLS content of the control group was 12.7 μmol/100 g on the 9th day, which dropped sharply to 4.2 μmol/100 g on the 15th day. However, the declining trend of the treatment group was not obvious. In the late storage period (12 d and 15 d), the TGLS content of fresh-cut “Xuebai” cauliflowers in the three treatment groups was higher than that in the CK group, showing a significant difference (*p* < 0.05). Treatment with temperature and CaCl_2_ can effectively delay the degradation of TGLS in fresh-cut “Xuebai” cauliflower tissues and maintain the good nutritional quality of cauliflowers. The content of TGLS in the group treated with CaCl_2_ + 40 °C was the highest. This shows that the effect of the 40 °C thermal compound treatment was better than that of the 0 °C and 20 °C treatments.

Glucosinolates (GLS) are natural biologically active ingredients in broccoli. The endogenous plant enzyme myrosinase in the plant cell, physically segregated from GLS, is released when chopped or chewed to hydrolyze GLS into various products that help prevent cancer, including isothiocyanates (ITCs), thiocyanates, nitriles, and sulforaphane [35]. GLS are distributed in the vacuoles of plants in the form of salts. In the early stages of storage, the increase in GLS due to the presence of hydroxycinnamic acid helps to maintain the rigidity of the cell walls, which can protect plant tissues from damage. However, in later stages of storage, a decrease in TGLS content occurs due to the degradation of GLS [19]. A.P. Vale et al. (2015) reported that the GLS content of broccoli during cold storage increased first and then decreased. The best consumption time was seven days after harvest. During this period of time, the aliphatic GL of broccoli was at a relatively high level. Sulforaphane was the major decomposition product, and it had the effect of reducing the risk of cancer [36]. Heat treatment can significantly improve the retention of glucoraphanin, maintain the quality of broccoli, and extend the shelf life of products [37]. A study on the effect of CaCl_2_ treatment on the GLS metabolism of broccoli sprouts showed that it promoted the expression of BrST5b (sulfotransferase 5b), inhibited AOP2 (2-oxoglutarate-dependent dioxygenase 2) expression, and induced the expression of genes that synthesize GLS to promote GLS biosynthesis [17].

#### 3.1.6. Polygalacturonase Activity

**Figure 5:**
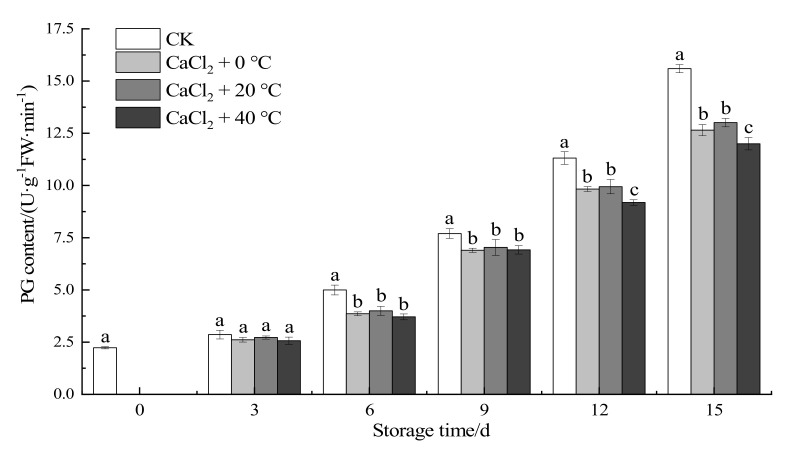
Effect of the different treatments on the PG activity of fresh-cut “Xuebai” cauliflower florets. Error bars indicate standard deviation (SD); the statistical significance between the four groups that passed the Tukey significance test is expressed in different lowercase letters, and the significance level is *p* < 0.05.

Polygalacturonase (PG) is an enzyme that is closely related to softening senescence, and plays an important role in the degradation of cell wall materials [38]. It can be seen from Figure 5 that, as the storage period grew longer, the activity of PG increased, and the activity of PG in the treatment groups was significantly lower than that in the CK group. There was no significant difference between the treatment groups and the control group in the early stage of the storage period (*p* > 0.05). In the late storage period, the PG activity of the fresh-cut “Xuebai” cauliflower florets in the treatment groups was lower than that in the control group, and there was a significant difference (*p* < 0.05). On the 15th day, the PG activity of the group given the CaCl_2_ + 40 °C treatment was 11.99, which was 23.1% lower than that of the control group. There was a significant difference in the PG activity between the group given the CaCl_2_ + 40 °C treatment and the other two treatment groups. The results showed that the treatment with temperature and CaCl_2_ compound effectively inhibited the activity of PG enzyme during storage and delayed the softening of fresh-cut “Xuebai” cauliflower florets.

During the maturation and senescence of fruits and vegetables, the activity of PG, which is a cell-wall-degrading enzyme, increases. As a result, cell wall components such as pectin are degraded, resulting in the softening of the pulp, increased juice yield, and changes in the textural characteristics. During storage, the de-esterification and long-chain depolymerization of pectin occur, leading to the degradations of insoluble pectin into soluble pectin and pectic acid. The adhesion between the cells is reduced, the cell partitions disappear, and the turgor pressure is lost. This causes the cell viscosity and fruit hardness to decrease and finally leads to the softening of the fruit [39]. Ca+ can interact with pectin to form a calciumpectin gel, causing the cell walls to harden and giving them the ability to resist degradation. The use of calcium reduces the accessibility of cell-wall-degrading enzymes [32], thereby causing the activity of PG enzyme to be lower in the treatment group than in the control group. In general, the inhibition achieved by the CaCl_2_ + 40 °C treatment was better than that achieved by the other two treatments. We also found that low temperatures do not effectively inhibit the activity of PG enzyme, which is consistent with the results of Tian et al. (2008) [38].

#### 3.1.7. Lipoxygenase Activity

**Figure 6:**
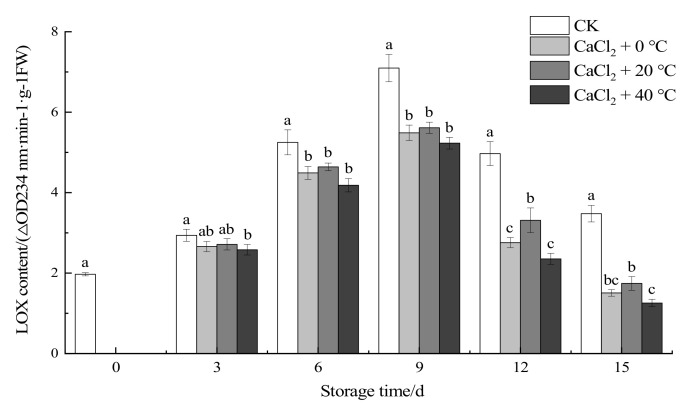
Effect of the different treatments on the LOX activity of fresh-cut “Xuebai” cauliflower florets. Error bars indicate standard deviation (SD); the statistical significance between the four groups that passed the Tukey significance test is expressed in different lowercase letters, and the significance level is *p* < 0.05.

Lipoxygenase (LOX) has a peroxidation effect on lipids. It can cause a large number of free radicals to be produced and participate in the synthesis of ethylene, leading to an increase rate of aging in fruits and vegetables [40]. Figure 6 shows that, with the increase in the storage period, the activity of LOX first increased and then decreased, while the LOX value of the treatment groups was lower than that of the CK group. On the third day, there was a significant difference between the fresh-cut “Xuebai” cauliflower florets in the three treatment groups and those in the CK group (*p* < 0.05). On the ninth day, the LOX enzyme activity of the control group and the treatment groups reached a peak, after which the cauliflower began to show rapid softening and worsening effects of aging. By the 15th day, there was a significant difference between the CaCl_2_ + 40 °C treatment group and the other two treatment groups (*p* < 0.05). It was observed that the CaCl_2_ + 40 °C treatment inhibited the LOX activity most significantly.

LOX can catalyze the oxidative metabolism of unsaturated fatty acids to generate lipid peroxidation free radicals and other substances, activate membrane lipid peroxidation, and aggravate the damage to the membrane structure. The combination of Ca+ and membranes can protect membrane lipids, reduce plant cell membrane damage, and strengthen the cell wall structure. Ca+ plays an important role in stabilizing the membrane lipid structure, preventing membrane damage and maintaining the membrane integrity. However, exposure to low temperatures will induce Ca+ exudation, increase the Ca+ content in the cytoplasmic matrix, increase the activity of lipoxygenase enzymes in the cell, and aggravate the degradation and peroxidation of membrane lipids [41]. These findings are consistent with our conclusion that the CaCl_2_ + 40 °C treatment was better than the CaCl_2_ + 0 °C treatment in this study.

### 3.2. Factor Analysis among Cauliflower Quality Indicators

We carried out an exploratory factor analysis with dimensionality reduction to extract and synthesize the original variables with overlapping information into a small number of common factors, thereby replacing most of the original variable information. This method was used to model and analyze the quality of fresh-cut “Xuebai“ cauliflower florets [42,43,44].

#### 3.2.1. Correlation Analysis of Quality Indicators

During storage, the cell structures of fresh-cut “Xuebai” cauliflower florets were damaged, the permeability of the cell membrane was increased, and the rigidity of the cell wall was decreased. With the degradation of insoluble pectin into soluble pectin and pectic acid, the firmness of fresh-cut “Xuebai” cauliflower florets decreased rapidly and the activity value of the PG enzyme showed an upward trend. However, the ascorbic acid content and L* value both showed downward trends. From the changing trends of the quality indicators of fresh-cut “Xuebai” cauliflower florets, it could be seen that there was an intrinsic connection between them, so it was necessary to carry out relationship research.

Table 2 shows the results of the relationship analysis carried out on the quality indicators of fresh-cut “Xuebai” cauliflower florets. Very significant positive relationships were observed between the firmness (X1) and L* value (X2), ASA (X5), TGLS (X6), LOX activity (X8), and SE (X9) (*p* < 0.01). Additionally, negative relationships could be clearly observed between the firmness (X1) and a* value (X3), b* value(X4), and PG activity (X7) (*p* < 0.01). We also observed extremely significant positive relationships between the L* value (X2) and ASA (X5) and TGLS (X6) (*p* < 0.01), as well as an extremely significant negative relationship between the a* value (X3) (*p* < 0.01) and PG (X7) (*p* < 0.05). The a* value (X3) had very significant positive relationships with the b* value (X4) and PG activity (X7) (*p* < 0.01). There was also a clear negative relationship between the a* value (X3) and ASA (X5), TGLS (X6), LOX activity (X8), and SE (X9) (*p* < 0.01). The b* value (X4) was extremely negatively associated with the ASA (X5) and TGLS content (X6) (*p* < 0.01), and significantly negatively associated with the LOX activity (X8) (*p* < 0.05). The content of ASA (X5) had a very significant positive relationship with the LOX activity (X8) and SE (X9) (*p* < 0.01). The content of TGLS (X6) had very significant positive relationships with the LOX activity (X8) and SE (X9) (*p* < 0.01). There were also very significant negative relationships between the PG activity (X7) and LOX activity (X8) and SE (X9) (*p* < 0.01).

From Table 2, it can be seen that the correlations between the quality indicators of fresh-cut “Xuebai” cauliflower florets were relatively high. However, if a quality assessment is carried out directly, there will be deviations in the assessment results due to the presence of overlapping information [45]. Thus, a factor analysis was conducted to combine several relevant indicators with uncorrelated indicators [44]. This new set of quality indicators was used for the quality synthesis of fresh-cut “Xuebai” cauliflower florets treated with different temperatures and CaCl_2_ in order to improve the reliability of the evaluation model.

#### 3.2.2. Principal Component Analysis (PCA)

The raw data of various indexes of fresh-cut “Xuebai” cauliflower florets treated with different temperatures and CaCl_2_ were statistically analyzed using the Kaiser-Meyer-Olkin (KMO) and Bartlett test methods. It can be seen from Table 3 that the KMO value was 0.794, indicating that the sampling adequacy effect was very good. The significant difference of Bartlett’s spheroid test was less than 0.01; this showed that the quality index data of fresh-cut “Xuebai” cauliflower florets were different. We continued to study these data with a factor analysis.

A principal component analysis (PCA) was carried out in the SPSS 17.0 software to extract feature values from the quality index data of fresh-cut “Xuebai” cauliflower florets and perform a factor analysis. Table 4 shows that the variances of the common factors of all indexes of fresh-cut “Xuebai” cauliflower florets were above 0.917. This indicated that the effect of the factors analysis was good, and that the quality index data of fresh-cut “Xuebai” cauliflower florets were suitable for factor analysis.

The PCA and the quarter rotation method were used to obtain the eigenvalues and rates of contribution of each factor of fresh-cut “Xuebai” cauliflower florets; the results are shown in Table 5.

Table 5 shows the variance contribution rate obtained in the factor analysis of fresh-cut “Xuebai” cauliflower florets. The contribution rates of factor one and factor two to the total variance of fresh-cut “Xuebai” cauliflower floret reached 85.744% and 11.768%, respectively, while the cumulative variance contribution rate reached 97.513%. Nearly 100% of the information in the original dataset was covered by these two principal components, so it was determined that the number of factors in the subsequent factor analysis should be two.

**Figure 7:**
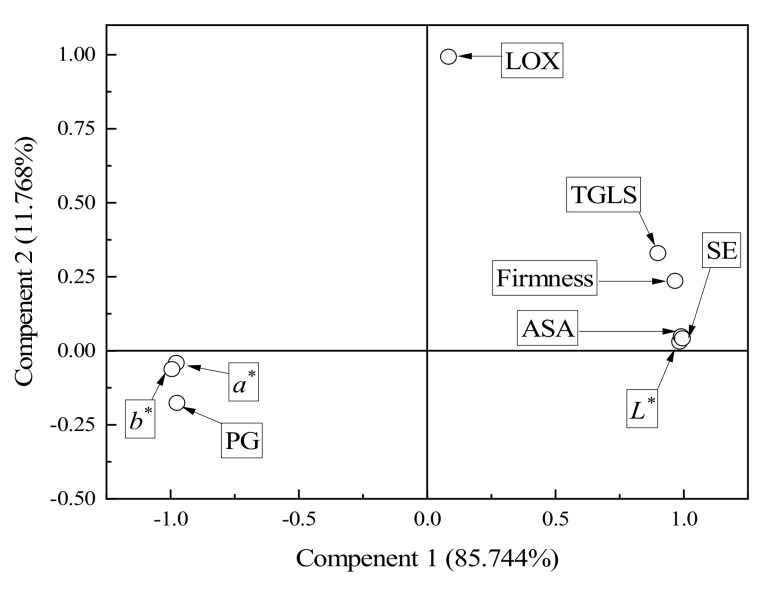
Principal component graph in rotating space. L*—lightness, a*—redness, b*—yellowness.

Figure 7 shows a schematic diagram of the factor rotation of the correlation coefficients of the nine original indicators and factor one and factor two. It can be seen from Figure 7 that firmness, TGLS, ASA, L* value, and SE had higher loads in the positive direction of factor one, while PG, a* value, and b* value had higher loads in the negative direction of factor one, which is in line with the actual meaning of each index. It was found that the higher the PG activity, a* value, and b* value were, the more significant the deterioration in the quality of fresh-cut “Xuebai” cauliflower florets was. Additionally, the higher the firmness, TGLS, ASA, L* value, and SE were, the higher the quality of fresh-cut “Xuebai” cauliflower florets was. It can be inferred that factor one comprehensively reflected the main quality indicators, while factor two had a significant positive correlation with the LOX content. This mainly demonstrates the influence of LOX on the storage quality of fresh-cut “Xuebai” cauliflower florets, and its cumulative variance contribution rate was only 11.768%. The impact of this factor on the overall quality was far less important than that of factor one.

Table 6 shows the effectiveness of the use of the regression coefficient method to explore the mutual effect of fresh-cut “Xuebai” cauliflower floret quality indicators under CaCl_2_ treatments at different temperatures. After the data were rotated using the maximum variance method, the expressions of factor one and factor two were obtained.

Y1 = 0.123 × X1 + 0.132 × X2 − 0.131 × X3 − 0.133 × X4 + 0.133 × X5 + 0.111 × X6 − 0.126 × X7 − 0.020 × X8 + 0.133 × X9(1)

Y2 = 0.091 × X1 − 0.106 × X2 + 0.096 × X3 + 0.079 × X4 − 0.090 × X5 + 0.189 × X6 − 0.032 × X7 + 0.933 × X8 − 0.098 × X9(2)

These two common factors reflected the effects of different treatments on the storage quality of fresh-cut “Xuebai” cauliflower florets in terms of different aspects. Then, the variance contribution rate corresponding to each common factor was weighted to obtain the following comprehensive score calculation Equation Y:

Y = (85.744 × Y1 + 11.768 × Y2) ÷ 97.513 = 0.119 × X1 + 0.103 × X2 − 0.104 × X3 − 0.107 × X4 + 0.106 × X5 + 0.120 × X6 − 0.115 × X7 + 0.095 × X8 + 0.105 × X9(3)

We normalized the original data and substituted them into Equation Y. Table 7 lists the order of the comprehensive quality index values of fresh-cut “Xuebai” cauliflower florets treated with different temperatures and CaCl_2_.

It can be seen from the comprehensive score comparison of CaCl_2_ treatments at different temperatures in Table 7 that the storage period of fresh-cut “Xuebai” cauliflower florets could be significantly prolonged after treatment with temperature and CaCl_2_. The fresh-cut “Xuebai” cauliflower florets had the highest score after being treated with CaCl_2_ + 40 °C, but as the storage time grew longer, their quality continued to decline. After 15 days of storage, the comprehensive quality score of the cauliflowers fell below the pass line and the curd softened and browned, causing then to lose their storage value.

## 4. Conclusions

This experiment studied the effect of the use of different temperatures and CaCl_2_ treatments on the preservation of fresh-cut “Xuebai” cauliflower florets. Through a comprehensive evaluation of our factor analysis, the optimal processing method for cauliflowers subjected to a storage period was determined. The test results showed that as the storage period grew longer, the fresh-cut “Xuebai” cauliflower florets developed a peculiar smell; darkened and yellowed in color; exhibited decreased hardness; decreased SE, ASA, and TGLS content, and showed increased PG and LOX activities. The quality of cauliflowers in the treatment groups was significantly better than that of the cauliflowers in the CK group, and they maintained a higher firmness value, higher ASA and TGLS content, and a lower activity of related enzymes. The factor analysis of indicators of fresh-cut “Xuebai” cauliflower florets during the storage period found that the rate of contribution of the first two principal component factors was 97.513%. These two factors were able to represent the change in the cauliflowers’ quality. We also established a comprehensive evaluation model for fresh-cut “Xuebai” cauliflowers. The CaCl_2_ + 40 °C treatment was determined to be the best preservation method for fresh-cut “Xuebai” cauliflower florets. This study provides a theoretical reference for extending the storage time of fresh-cut “Xuebai” cauliflower florets and improving their quality during storage.

## Figures and Tables

**Table 1:** Effects of different treatments on the color of fresh-cut “Xuebai” cauliflower florets.

Color	Groups		Storage Time/d				
		0	3	6	9	12	15
L*	CK	70.265 ± 0.96 a	69.207 ± 0.961 a	67.883 ± 0.744 a	66.127 ± 0.736 b	63.467 ± 0.652 c	59.923 ± 0.549 c
	CaCl_2_ + 0 °C		69.647 ± 0.806 a	67.810 ± 0.902 a	67.510 ± 0.711 ab	66.073 ± 0.347 ab	64.263 ± 0.346 b
	CaCl_2_ + 20 °C		69.423 ± 0.612 a	68.463 ± 0.728 a	67.290 ± 0.651 ab	65.840 ± 0.517 b	63.733 ± 0.481 b
	CaCl_2_ + 40 °C		69.873 ± 0.849 a	69.240 ± 0.731 a	68.360 ± 0.580 a	67.370 ± 0.723 a	65.700 ± 0.637 a
a*	CK	−1.558 ± 0.67 a	−1.517 ± 0.061 a	−1.457 ± 0.098 a	−1.353 ± 0.057 a	−1.187 ± 0.051 a	−0.987 ± 0.080 a
	CaCl_2_ + 0 °C		−1.540 ± 0.062 a	−1.503 ± 0.062 a	−1.450 ± 0.053 a	−1.357 ± 0.032 b	−1.283 ± 0.049 b
	CaCl_2_ + 20 °C		−1.533 ± 0.081 a	−1.490 ± 0.070 a	−1.427 ± 0.031 a	−1.333 ± 0.091 b	−1.220 ± 0.123 b
	CaCl_2_ + 40 °C		−1.547 ± 0.076 a	−1.517 ± 0.100 a	−1.467 ± 0.055 b	−1.397 ± 0.060 b	−1.317 ± 0.075 b
b*	CK	9.61 ± 0.35 a	10.927 ± 0.787 a	12.877 ± 0.962 a	14.840 ± 0.792 a	17.413 ± 0.751 a	20.317 ± 0.587 a
	CaCl_2_ + 0 °C		10.433 ± 0.656 a	11.690 ± 0.797 a	12.977 ± 0.290 b	15.393 ± 0.562 b	16.677 ± 0.621 bc
	CaCl_2_ + 20 °C		10.480 ± 0.579 a	11.877 ± 0.670 a	13.347 ± 0.696 b	15.277 ± 0.535 b	17.430 ± 0.622 b
	CaCl_2_ + 40°C		10.260 ± 0.321 a	11.470 ± 0.810 a	12.797 ± 0.597 b	14.547 ± 0.621 b	16.100 ± 0.085 c

Note: Different letters in the same column indicate significant differences at the *p* < 0.05 level. L*—lightness, a*—redness, b*—yellowness.

**Table 2:** Relationship analysis of the quality indicators of fresh-cut “Xuebai” cauliflower florets during storage.

	Firmness	L* (X2)	a* (X3)	b * (X4)	ASA (X5)	TGLS (X6)	PG (X7)	LOX (X8)	SE (X9)
Firmness (X1)	1.000								
L* (X2)	0.957 **	1.000							
a* (X3)	−0.949 **	−0.995 **	1.000						
b* (X4)	−0.976 **	−0.981	0.978 **	1.000					
ASA (X5)	0.973 **	0.961 **	−0.950 **	−0.991 **	1.000				
TGLS (X6)	0.930 **	0.880 **	−0.877 **	−0.900 **	0.899	1.000			
PG (X7)	−0.988 **	−0.943 *	0.936 **	0.981	−0.985	−0.935	1.000		
LOX (X8)	0.316 **	0.120	−0.131 **	−0.148 *	0.130 **	0.381 **	−0.251 **	1.000	
SE (X9)	0.972 **	0.968	−0.965 **	−0.995	0.993 **	0.899 **	−0.986 **	0.125	1.000

* and ** represent significance at the levels of 0.05 and 0.01. L*—lightness, a*—redness, b*—yellowness.

**Table 3:** KMO and Bartlett’s correlation test.

Test Method	KMO Measure of Sampling Adequacy	Bartlett’s Test of Sphericity		
		Approx. x2	df	Sig.
Result	0.794	477.288	36	0.000

**Table 4:** Factor variance statistics of quality indicators.

Quality Indexes	Initial	Extraction
Firmness(X1)	1.000	0.989
L*(X2)	1.000	0.969
a*(X3)	1.000	0.959
b*(X4)	1.000	0.994
ASA(X5)	1.000	0.983
TGLS(X6)	1.000	0.917
PG(X7)	1.000	0.981
LOX(X8)	1.000	0.993
SE(X9)	1.000	0.991

L*—lightness, a*—redness, b*—yellowness.

**Table 5:** Factor analysis of principal component characteristic values and contributions in fresh-cut “Xuebai” cauliflower florets.

Component	Initial Eigenvalues			Extraction Sums of Squared Loadings			Rotation Sums of Squared Loadings		
	Total	% of Variance	Cumulative	Total	% of Variance	Cumulative	Total	% of Variance	Cumulative
1	7.736	85.960	84.960	7.736	85.960	84.960	7.717	85.744	85.744
2	1.040	11.553	97.513	1.040	11.553	97.513	1.059	11.768	97.513
3	0.113	1.261	98.773						
4	0.085	0.949	99.722						
5	0.013	0.141	99.863						
6	0.008	0.086	99.949						
7	0.002	0.025	99.974						
8	0.002	0.017	99.991						
9	0.001	0.009	100.000						

**Table 6:** Quality component coefficient matrix of fresh-cut “Xuebai” cauliflower florets.

Quality indexes	Component	
	1	2
Firmness (X1)	0.123	0.091
L* (X2)	0.132	−0.106
a* (X3)	−0.131	0.096
b* (X4)	−0.133	0.079
ASA (X5)	0.133	−0.090
TGLS (X6)	0.111	0.189
PG (X7)	−0.126	−0.032
LOX (X8)	−0.020	0.933
SE (X9)	0.133	−0.098

L*—lightness, a*—redness, b*—yellowness.

**Table 7:** Ranking of the comprehensive quality index values of fresh-cut “Xuebai” cauliflower florets treated with different temperatures and CaCl_2_.

Method	Storage Time/d	Value	Rank
CK	3	103.285	4
	6	99.066	8
	9	89.918	12
	12	78.230	16
	15	60.603	20
CaCl_2_ + 0 °C	3	104.983	2
	6	101.324	6
	9	94.059	10
	12	84.302	14
	15	70.889	18
CaCl_2_ + 20 °C	3	104.545	3
	6	100.455	7
	9	93.628	11
	12	83.878	15
	15	70.091	19
CaCl_2_ + 40 °C	3	105.410	1
	6	102.522	5
	9	96.307	9
	12	87.251	13
	15	74.803	17

## Data Availability

Not applicable.
